# Seasonal Variation of Arsenic Concentrations in Tubewells in West Bengal, India

**Published:** 2006-09

**Authors:** Xavier Savarimuthu, Meera M. Hira-Smith, Yan Yuan, Ondine S. von Ehrenstein, Subhankar Das, Nilima Ghosh, D.N. Guha Mazumder, Allan H. Smith

**Affiliations:** ^1^ Kalyani University, Kalyani, West Bengal, India; ^2^ Arsenic Health Effects Research Group, School of Public Health, University of California-Berkeley, Berkeley, CA 94720, USA; ^3^ Institute of Post-Graduate Medical Education and Research, Kolkata 700 020, India

**Keywords:** Arsenic, Arsenic contamination, Tubewell, Seasonal variations, Water pollution, Drinking-water, Water supply, India

## Abstract

This study was conducted to monitor the changes in arsenic concentration during different seasons in a one-year period during 2002–2003 in selected tubewells in an arsenic-affected area in the district of South 24 Parganas in West Bengal, India, and to map the location of the wells. Seasonal variations in concentrations of arsenic in water were measured from 74 selected tubewells, ranging in depth from 40 to 500 feet. Water samples were collected from these wells during winter, summer, monsoon, and the following winter in 2002–2003. A global positioning system was used for locating the tubewells, and a geographic information system was used for mapping. There was evidence of seasonal variation in concentrations of arsenic in water (p=0.02) with the minimum average concentration occurring in the summer season (694 μg/L) and the maximum in the monsoon season (906 μg/L). From the winter of 2002 to the winter of 2003, arsenic concentrations increased, irrespective of the depth of the tubewells, from an average of 464 μg/L to 820 μg/L (p<0.001). This extent of variation in arsenic concentration, if confirmed, has important implications for both epidemiological research and mitigation programmes.

## INTRODUCTION

Knowledge of the extent of arsenic contamination of groundwater is increasing rapidly. In 1918, Ayerza described the impact of arsenic in drinking-water on human health in Argentina (1). In Asia, the countries where skin lesions caused by arsenic in drinking-water have been reported extensively in recent years. These countries are: India, Nepal, Pakistan, Myanmar, Taiwan, Viet Nam, Laos, part of China, including Inner Mongolia, Thailand, Cambodia, and Bangladesh (2–4). In all these regions, the main source of arsenic in drinking-water is sediment of the quaternary period that contains considerable amounts of arsenic. Arsenic is mobilized from the soil to the water by complex geochemical mechanisms (5).

Evidence suggests the presence of arsenic in groundwater in India and Bangladesh throughout the region defined as the Indo-Gangetic Plain (4). The lower part of the Ganges basin lies in the state of West Bengal where 9 of 18 districts are within the area identified as containing elevated concentrations of arsenic in groundwater above 50 μg/L (4). The potential variation in concentrations of arsenic in well-water over time has received little attention. This is important for investigations of health effects relating to concentrations of arsenic in tubewells and also for mitigation, since tubewells thought to contain safe water at one point in time may need to be periodically monitored. The aim of this study was to monitor the change in arsenic concentration during different seasons in a one-year period during 2002–2003 in selected tubewells in an arsenic-affected area in West Bengal, India, and to map the location of the wells. Tubewells which had arsenic concentrations of above 100 μg/L were selected because high concentrations of arsenic and the accuracy of their assessment are important to studies of health effects, including identifying dose-response relationships.

## MATERIALS AND METHODS

**Fig. 1.**
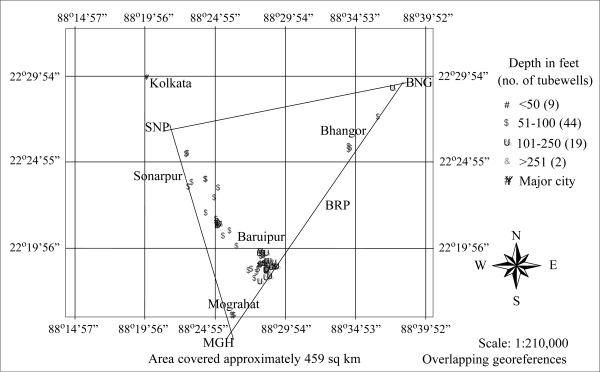
Georeferences of tubewells by depth and total area covered for seasonal variations in South 24 Parganas

This investigation was conducted in the arsenic-affected area of the district of South 24 Parganas in West Bengal, India, which was selected previously to conduct a series of studies on the health effects of arsenic (6–8). The total population of the district is 6.9 million with a population density of 694 persons per sq km (9). The district, with an area of 9,960 sq km, is subdivided into 29 smaller areas, called Police Stations. Four Police Stations, namely Sonarpur (SNP), Baruipur (BRP), Bhangor (BNG), and Mograhat (MGH), were selected for the present study. The total area traversed in the study was approximately 459 sq km, which is 5% of the total area of the district (Fig. 1). The main sources of drinking-water for the people in the rural areas are private or government tubewells, tapping water from shallow aquifers ranging from 50 to 200 feet. There are also some deep tubewells in the area which generally have low concentrations of arsenic. However, samples from some tubewells deeper than 500 feet showed arsenic levels of above 50 μg/L (10).

BNG=Bhangor;
BRP=Baruipur;
MGH=Mograhat;
SNP=Sonarpur

A portable Garmin E-trex GPS device was used for GIS mapping and for assisting in relocating the tubewells. The georeferences—latitude and longitude—were recorded in decimal degrees, and the waypoints were stored in the device until transferred to the computer with G7ToWin software. Data were saved as CSV (comma separated value) files that automatically convert to spread-sheets and were saved as dBase IV to be used by the Arc View software. The Arc View software version 3.2 was used for location of the tubewells, graduated colour-codes denoting the tubewells by depth and the number of tubewells in each Police Station in the seasonal variations study.

Tubewells for the seasonal variability study were selected based on the results of arsenic analysis at the Institute of Post-Graduate Medical Education and Research (IPGME&R), Kolkata, by flow-injection hydride-generation atomic absorption spectrophotometry (AAS) (11). The detection limit for the IPGME&R was close to 2 μg/L based on an inter-laboratory cross-referencing programme which was undertaken with the University of Washington Laboratory of Professor David Kalman. Eight hundred water samples were collected for earlier arsenic health-effect studies (12, 13). We selected 74 of these tubewells with arsenic concentrations of >100 μg/L for this study. The georeferences of the tubewells were recorded as depicted in Figure 1. Analyses of arsenic for seasonal variation samples were conducted at the IPGME&R using the same flow-injection hydride-generation AAS referred to above. Water samples were collected in seasons as follows: winter 2002 (December 2002—February 2003), summer (end of April 2003—mid-May), monsoon (end of July—mid-September), and winter 2003 (mid-December 2003—January 2004). In the first winter, samples were obtained from 65 of the 74 tubewells, but all 74 tubewells were sampled during the following three seasons. For this reason, the seasonal analysis involved the summer, monsoon and second winter samples from the 74 tubewells, while the analysis for the change in concentrations one year apart was assessed for the 65 tubewells sampled in the winters of 2002 and 2003. The maximum depth of the tubewells was 500 feet, and the minimum was 40 feet. The tubewells are depicted by depth in Figure 1.

### Statistical methods

The mean concentration of arsenic was calculated first after stratifying by seasons and years, then after further stratifying by depth of tubewells. Repeated measures of analysis of variance (ANOVA) (14) were conducted to determine if arsenic concentration varied within 74 wells over three seasons in 2003 and if arsenic concentration changed in 65 wells between winter 2002 and winter 2003. In the seasonal analysis, we tested for the main effects of season and depth, and season-depth interaction. In the winter-to-winter analysis, we tested for main effects of year and depth and year-depth interaction. We conducted all analyses using SAS PROC GLM (15). The repeated measures of analysis of variance take into account that water samples are obtained from the same tubewells during each season. We have not depicted variation in individual wells because our hypotheses relate to tubewells in the aggregate.

## RESULTS

**Fig. 2.**
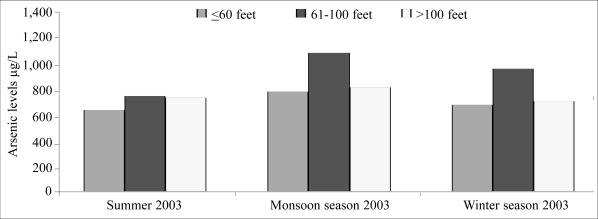
Seasonal variations of average arsenic concentrations with standard errors of the means by depth in South 24 Parganas

Table 1 and Figure 2 present the findings of seasonal analyses. There was evidence for seasonal variation with the minimum average concentration occurring during the summer season (694 μg/L) and the maximum during the monsoon when the average was 906 μg/L (test for seasonal variation by repeated measures of analysis of variance, p=0.02). There was a wide variation between wells with 28 (38%) having arsenic levels of monsoon season more than 25% higher than the average of the measurements for the other two seasons, but 17 (23%) wells showing the opposite trend with concentrations of 25% or lower during the monsoon season. There was no evidence for variation by depth of tubewell (p=0.3) and also no evidence for an interaction between the depth of the wells and season (p=0.4).

**Table 1.** Arsenic concentrations by depth of tubewells in three seasons

Depth (feet) of tubewells	No. of tubewells	Mean (standard deviation) arsenic concentration (μg/L)		
		Summer 2003	Monsoon 2003	Winter 2003
≤60	21	622 (486)	766 (842)	660 (585)
61–100	33	729 (338)	1063 (898)	940 (702)
>100	20	714 (418)	796 (646)	693 (580)
All	74	694 (403)	906 (822)	794 (644)

Table 2 presents the findings from one year to the next. Arsenic concentrations were much higher in the winter of 2003 (average 820 μg/L) than in the winter of 2002 (average 464 μg/L) (p<0.001). There was no evidence that the increase varied according to the depth of wells (p=0.14), nor was their interaction between depth and year (p=0.21). There was a wide variation between wells with 45 (69%) having arsenic levels of 25% or more higher in the winter of 2003 than in the winter of 2002, but eight (12%) wells demonstrated the opposite trend with levels of more than 25% lower in the winter of 2003 than in 2002.

**Table 2.** Arsenic concentrations by depth of tubewells in winter in 2002 and 2003

Depth (feet) of tubewells	No. of tubewells	Mean (standard deviation) arsenic concentration (μg/L)	
		Winter 2002	Winter 2003
≤60	19	375 (288)	635 (584)
61–100	30	517 (363)	996 (712)
>100	16	472 (298)	708 (607)
All	65	464 (328)	820 (663)

## DISCUSSION

The results of this investigation were unexpected since we had assumed that concentrations of arsenic in tubewells would be relatively stable over time and did not expect to find much seasonal variation, if any. We have previously measured correlation coefficients between arsenic concentrations measured in wells in Nevada, USA, over 1–5, 6–10 and 11–20 years apart. The correlation coefficients were 0.84 (95% confidence interval [CI] 0.81–0.86), 0.85 (95% CI 0.81–0.88), and 0.94 (95% CI 0.88–0.96) (16). The increase in concentrations of arsenic in water between the two winter seasons in West Bengal was surprising. We do not have a good explanation for this, although there was a remarkable difference in monsoon rainfall between 2002 (1,978 mm) and 2003 (1,441 mm) (17). The lower rainfall in the monsoon of 2003 might be related to higher concentration of arsenic in tubewell water in following winter, although we do not have a mechanism to explain this.

Regarding seasonal variation, the maximum concentrations of arsenic in tubewell water were present in samples taken during the monsoon of 2003 (Fig. 2). There is limited information on seasonal variation of arsenic concentration in wells in previous studies. One study used peizometers to investigate the mechanism of movement of arsenic in groundwater in West Bengal (18). Wells were sampled on three occasions over 18 months. The authors stated that “no changes were seen in concentrations of arsenic”, but they did not present the data.

In Bangladesh, a study was conducted in Araihazar which included monitoring of arsenic concentrations in 14 tubewells (19). The report stated that “over the course of a year arsenic concentrations did not show any notable temporal fluctuations.” In one shallow tubewell, “some apparent increase in the monsoon period” was noted involving variation between 35 μg/L and 66 μg/L. In another study in Bangladesh, six tubewells with low concentrations of arsenic (<50 μg/L) were monitored over a one-year period (20). According to the authors, there was no indication of significant seasonal fluctuation in concentrations of arsenic in these six tubewells. A recent publication which appeared after this paper was first submitted involved a further study in Bangladesh in which 20 wells were monitored over a three-year period. The findings were mixed. One well showed higher concentrations during the wet season, and the authors postulated that “the rise in As [arsenic] concentrations during the wet season could be attributed to the local dissolution of iron oxyhydroxides as conditions become more reducing, while during the dry season As is sca-venged onto fresh iron oxyhydroxides”. In contrast to the present study of 74 tubewells in West Bengal having an average arsenic concentration of close to 700 μg/L, the concentrations of arsenic in the monitored wells in Bangladesh were all below 50 μg/L. It could be that the number of wells was insufficient to detect seasonal variation in the Bangladesh case, or that significant seasonal variation only occurs in highly-contaminated wells.

Possible long-term and seasonal variations in concentrations of arsenic in well-water have important implications for both epidemiological research and mitigation programmes. Epidemiological studies frequently require using current samples from wells to estimate past concentrations of arsenic in drinking water (12). Potential long-term and seasonal variations would, therefore, add to the uncertainty in exposure assessment, reduce the power of such studies to detect health effects, and would also reduce the precision of risk estimates. With regard to mitigation, if there is long-term or seasonal variations in concentrations, tubewells with low concentrations of arsenic in water which are continuing to be used need to be monitored over time to ensure that concentrations of arsenic in water of these tubewells remain at an acceptable level over time. Both these undesirable consequences of seasonal variation and variation over time in concentrations of arsenic in water suggest that further investigations are needed to confirm the current findings.

## ACKNOWLEDGEMENTS

The principal author (Xavier Savarimuthu) was a Trainee in the Fogarty International Research and Training Program in Environmental and Occupational Health, National Institutes of Health (Fogarty award no. D43-TW000815). Other support came from the National Institute of Environmental Health Sciences (grant no. P42-ES04705) and the University of California Center for Occupational and Environmental Health. The authors are indebted to the tubewell owners who allowed sampling the water from their wells. The authors also acknowledge the assistance given by Anath Pramanick in locating the tubewells.
